# Clinical outcomes and revision rates following four-level anterior cervical discectomy and fusion

**DOI:** 10.1038/s41598-022-09389-1

**Published:** 2022-03-29

**Authors:** Anastasios Charalampidis, Nader Hejrati, Hari Ramakonar, Pratipal S. Kalsi, Eric M. Massicotte, Michael G. Fehlings

**Affiliations:** 1 Department of Clinical Science, Intervention and Technology (CLINTEC), Karolinska Institutet, Stockholm, Sweden; 2 Department of Reconstructive Orthopaedics, Karolinska University Hospital, Stockholm, Sweden; 3 Division of Genetics and Development, Krembil Brain Institute, University Health Network, Toronto, ON Canada; 4 Division of Neurosurgery, Krembil Neuroscience Centre, Toronto Western Hospital, University Health Network, University of Toronto, 399 Bathurst St, Toronto, ON M5T2S8 Canada; 5 Division of Neurosurgery, Department of Surgery, University of Toronto, Toronto, ON Canada; 6 Imperial College Healthcare, NHS Trust Charing Cross Hospital, London, W6 8RF UK

**Keywords:** Medical research, Outcomes research

## Abstract

Studies on outcomes after four-level anterior cervical discectomy and fusion (ACDF) are limited in the literature. The purpose of this study was to report on clinical outcomes and revision rates following four-level ACDF. Patients operated with four-level ACDF were identified in a prospectively accrued single institution database. Outcome scores included the Neck Disability Index (NDI) and Visual Analogue Scale (VAS) for neck and arm pain. Reoperation rates were determined. Any complications were identified from a review of the medical records. Twenty-eight patients with a minimum of 12 months follow up were included in the analysis. The mean age at surgery was 58.5 years. The median radiographic follow up time was 23 (IQR = 16–31.25) months. Cervical lordosis was significantly improved postoperatively (− 1 to − 13, p < 0.001). At the median 24 (IQR = 17.75–39.50) months clinical follow up time, there was a significant improvement in the NDI (38 to 28, p = 0.046) and VAS for neck pain scores (5.1 to 3, p = 0.012). The most common perioperative complication was transient dysphagia (32%) followed by hoarseness (14%). Four (14%) patients required revision surgery at a median 11.5 (IQR = 2–51) months postoperatively. The results of this study indicate that patients who undergo four-level ACDF have a significant improvement in clinical outcomes at median 24 months follow up. Stand-alone four-level ACDF is a valid option for the management of complex cervical degenerative conditions.

## Introduction

Anterior cervical discectomy and fusion (ACDF) is one of the most commonly performed procedures in the cervical spine^1–3^. Since Smith and Robinson^4^ first introduced the procedure in 1958, ACDF has been widely accepted as a method to treat patients with symptomatic myelopathy and/or radiculopathy secondary to cervical spondylosis^5^.

While in 1- and 2-level pathologies ACDF has been shown to be a highly successful procedure associated with significant improvement in clinical outcomes and high fusion rates^6^, its role in patients with multilevel cervical spondylosis is not well investigated. Several studies have reported that ACDF involving many levels is associated with higher rates of pseudarthrosis and complications due to extensive soft tissue dissection as well as the increased bone surface area that needs to be fused^6–8^. Four-level ACDF, a less commonly performed procedure, is particularly lacking in data reporting on outcomes after surgery^9–17^.

The purpose of this study is to report on clinical outcomes and revision rates following stand-alone four-level ACDF at a single institution.

## Materials and methods

Following approval from the University of Health Network Research Ethics Board, a review of prospectively collected data from the Toronto Western Hospital Operative Database was performed to identify all patients with cervical spondylosis undergoing four-level ACDF between 2005 and 2019 at a single institution. All methods were carried out in accordance with the approved guidelines and patients provided informed consent prior to surgery. Patients with previous cervical spine surgery, those undergoing combined anterior/posterior approaches, those with more than one-level corpectomy and those with shorter than 12 months clinical follow-up were excluded.

Patient demographics including age, gender, and smoking status were collected. Perioperative surgical data included number of surgical levels, American Society of Anesthesiologists (ASA) Grade, operative time and estimated blood loss. Any complications were identified from a review of the medical records.

### Radiographic assessment

Each individual’s preoperative and postoperative radiographs were assessed by one of the authors not involved in the patient’s care. Postoperative radiographs were taken periodically in an outpatient setting to assess bony fusion and to assure the integrity of the material. The parameters examined were: (1) preoperative and postoperative cervical lordosis measured from C2 to C7 on lateral radiographs according to the Cobb method^18^ and (2) evidence of fusion on the last available radiograph. Radiographic evidence of fusion was considered present if the following features were observed: (1) no motion across the fusion site on flexion–extension X-rays, (2) trabeculae across the fusion site, or (3) no lucency across the fusion site or around any of the screw sites^19^.

### Patient-reported questionnaire

Patient reported outcome measures (PROMs) were collected prior to surgery (baseline) as well as at 3–6 months and 1–2 years after surgery. PROMs were periodically collected thereafter, depending on the clinical situation and need for further follow-up, as determined on a case-by-case basis. The last available PROMs were used in this analysis.

The patient reported questionnaire contained the Neck Disability Index (NDI)^20^ and the Visual Analogue Scale (VAS)^21^ for neck and arm pain.

The Neck Disability index (NDI)^20^, with its 10-item scaled questionnaire, is a modification of the Oswestry Disability Index (ODI)^22^. It is widely used as a self-reported questionnaire for assessment of disability in patients with neck pain. The total NDI score ranges from 0 (no disability) to 100 (maximal disability).

The Visual Analogue Scale (VAS)^21^ is one of the most common and widely used assessment tools in the measurement of pain. It ranges from 0 (no pain) to 10 (worst possible pain).

### Operative technique

Operative technique was standardized across the 2 surgeons. Patients were placed supine with the occiput resting on the donut and a bump placed transversely under the scapula providing appropriate neck extension. The shoulders were taped. Somatosensory-evoked potential and EMG monitoring were used in all cases. Motor evoked potential (MEP) monitoring was used on a select basis in higher risk cases with significant spinal cord compromise. A right-sided transverse incision was performed and the anterior cervical spine was exposed using a Smith-Robinson approach^4^. A thorough removal of all disc material using microscopic visualization, removal of cartilage with microsurgical curettes as well as decortication with high-speed burr and careful attention to not violate the endplates was performed. Pre-contoured lordotic machined allografts were used in most cases. In cases where a corpectomy was performed, fibula allograft was used as necessary. The operative vertebral bodies were spanned with a lordotic titanium plate in a locking fashion. A Jackson Pratt type drain was used in most cases.

### Statistical analysis

Results were presented as the median with Interquartile Range (IQR) and mean ± standard deviation, where applicable. A Mann–Whitney U test was used for group comparisons and a Wilcoxon signed-rank test was used for within group comparisons. Missing data was handled with case-by-case exclusion, using pairwise deletion in the analyses. Statistical significance was set at p < 0.05. IBM SPSS statistical software version 23 was used to perform statistical analyses.

## Results

### Patient demographics and surgeon-reported data

A total of 36 patients who underwent a four–level ACDF at C3–C7 were identified in the database; 28 with a minimum of 12 months clinical follow-up were included in the analysis. Baseline PROMs for the variables of interest were available for 18 patients; 16 had complete data sets and 2 patients had data sets with missing values. The mean age at surgery was 58.5 (± 11), ranging from 41 to 79 years. There were 15 (53%) males. Five (18%) patients were smokers. Fifteen (54%) patients were classified as ASA III and 10 (35%) ASA II. Of the 28 patients, 6 (21%) underwent a hybrid procedure with one level corpectomy. The mean operative time was 257 min (± 59) and the mean estimated blood loss was 134 ml (± 76) (Tables 1 and 2).

**Table 1:** Summary of baseline characteristics for patients undergoing four-level ACDF.

Variables N = 28	
Age at surgery, years	58.5 (11)
Male gender	15 (54)
Cervical spondylosis with symptomatic myelopathy	17 (61)
Cervical spondylosis with symptomatic axial pain +/− radiculopathy	11 (39)
**ASA grade**	
I	1 (4)
II	10 (35)
III	15 (53)
IV	1 (4)
Smokers	5 (18)

Descriptive data is presented as number (percentage) or mean (SD).

*ACDF* anterior cervical discectomy and fusion, *ASA* American Society of Anesthesiologists.

**Table 2:** Summary of perioperative data and complications for patients undergoing four-level ACDF.

N = 28	
Variables	
**Operated level**	
C3 to C7	28 (100)
**One level corpectomy**	6 (21)
C4	2
C5	3
C6	1
Estimated blood loss, ml	134 (76)
Operative time, min	257 (59)
**Complications (%)**	
Dysphagia	9 (32)
Hoarseness	4 (14)
C5 palsy	1 (3.6)
C7 nerve root injury	1 (3.6)
Other	2 (7)

Descriptive data is presented as number (percentage) or mean (SD).

*ACDF* anterior cervical discectomy and fusion.

**Figure 1:**
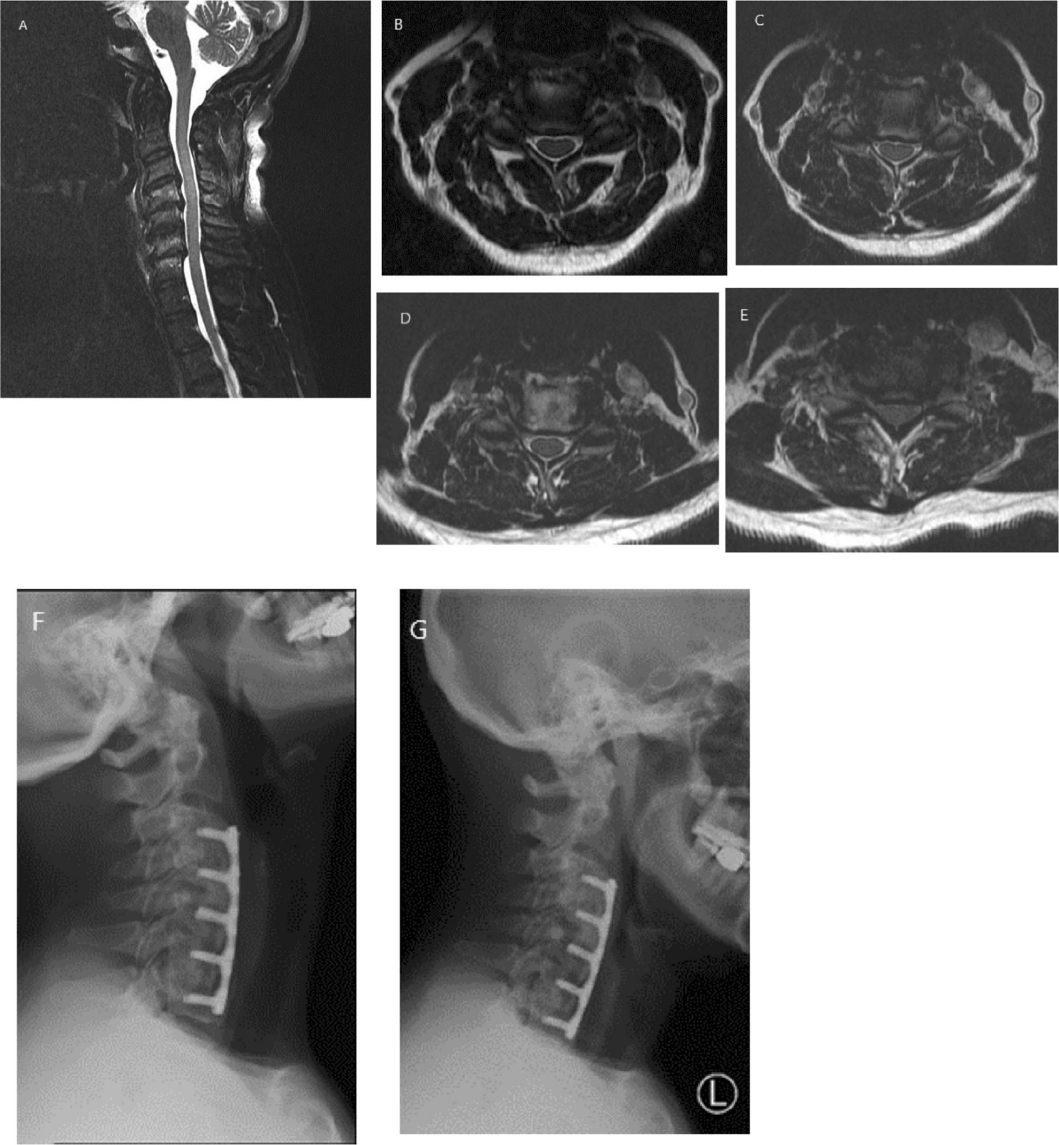
A 57-year-old female with longstanding history of neck pain. Over the past few years, she has also started to notice a gradual decrease in dexterity and numbness in her hands. T2-weighted sagittal and axial magnetic resonance imaging (MRI) scans (**A**–**E**) showed multilevel cervical stenosis at the interspace levels C3–C7. The patient underwent a four-level ACDF at the levels C3–C7 with excellent clinical outcome. At the last follow-up, lateral flexion/extension X-rays of the cervical spine (**F**,**G**) showed good alignment and solid fusion across each disc segment.

**Figure 2:**
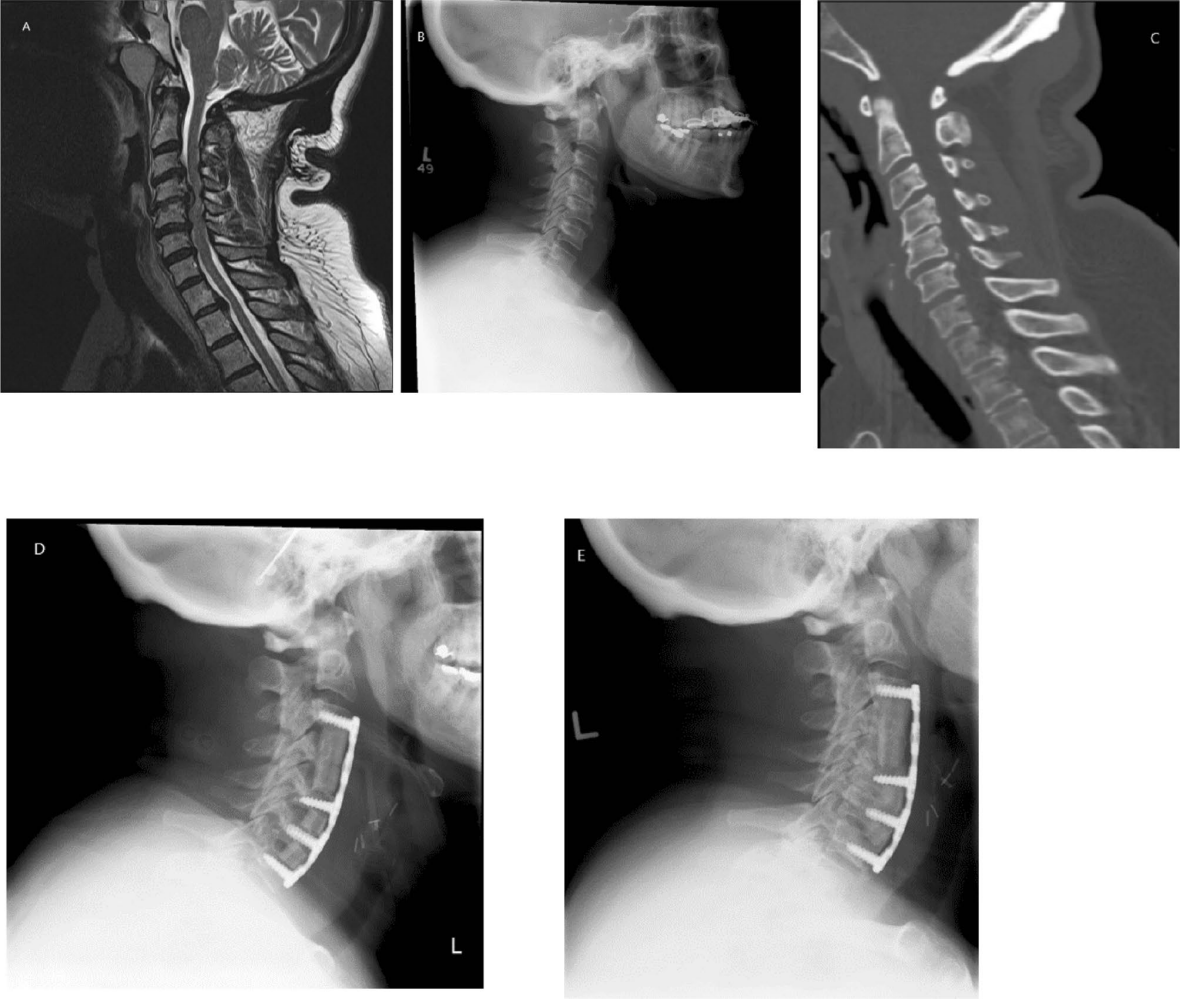
A 65-year-old female who presented with severe progressive cervical spondylotic myelopathy. MRI scan of the cervical spine showed multi-level cervical spondylosis with quite severe anterior cord compression, particularly at the C4/C5 level where there was a large partially sequestered disk (**A**). There was also significant spondylosis at C3/C4 and at C5/C6, and there was fairly significant degeneration of the foraminal narrowing at C6/C7 (**B**,**C**). The patient underwent a multilevel ACDF with C4 corpectomy (**D**). At the last follow-up 2 years postoperatively, X-rays of the cervical spine showed good alignment and solid fusion across each disc segment (**E**).

Figures 1 and 2 demonstrate two illustrative cases of patients undergoing four-level ACDF.

### Radiographic outcomes

The median radiographic follow-up was 23 months (IQR = 16–31.25), ranging from 5 to 144 months. At the median follow-up time, there was a significant improvement in cervical lordosis (− 1 to − 13, p < 0.001).

Four (14%) out of 28 patients were identified to have a pseudarthrosis. One patient was a smoker. For these 4 patients, the median radiographic follow-up was 17.5 months (IQR = 10.5–32), shorter compared to the median 23 months (IQR = 17–36.25) for the rest of the patients in the cohort. However, this difference was not significant (p = 0.4).

### Patient-reported outcomes

The median clinical follow up time was 24 months (IQR = 17.75–39.50), ranging from 12 to 94 months. At the median follow up time, there was a significant improvement in NDI scores (38 to 28, p = 0.046) and VAS for neck pain scores (5.1 to 3, p = 0.012). VAS for arm pain was also improved, however this improvement was not statistically significant (Table 3).

**Table 3:** Outcomes of all patients operated with four-level ACDF.

N = 28	Preoperative	Postoperative	Z	P
C2–C7 Cobb angle	− 1 (12)	− 13 (9)	− 4.109	** < 0.001**
NDI	38 (22.6)	28 (20.9)	− 1.993	**0.046**
VAS neck pain	5.1 (3.4)	3(2.7)	− 2.504	**0.012**
VAS arm pain	3.8 (3)	2.8 (3.2)	− 1.526	0.1

Descriptive data is presented as mean (SD or range). Bold values indicate statistical significance (P < 0.05).

*ACDF* anterior cervical discectomy and fusion, *NDI* neck disability index, *VAS* visual analogue scale.

### Perioperative complications

A summary of perioperative complications is presented in Table 2. The most common complication was transient postoperative dysphagia; it was observed in 9 (32%) patients. Spontaneous resolution of dysphagia was observed in all patients within 3–14 months after the surgery.

Postoperative hoarseness was observed in 4 (14%) patients. In 3 patients, symptoms had resolved within 3 months after surgery. One patient underwent vocal cord assessment 11 months after surgery due to persistent hoarseness; the assessment showed resolving post-intubation laryngeal granuloma. Hoarseness had resolved at the last follow up 22 months after surgery.

Postoperative C5 palsy with sensory loss and muscle weakness in the deltoid was observed in 1 (3.6%) patient. The patient made a full recovery at the last follow up 24 months after surgery. One patient (3.6%) sustained a right-sided C7 nerve root injury perioperatively that led to permanent sensory loss and motor deficit.

Other complications included: wound dehiscence in one case (3.6%) that was treated conservatively with antibiotics and Horner syndrome in one patient (3.6%) with complete resolution of symptoms 7 months postoperatively.

### Reoperation rate

Four patients (14%) required revision surgery at a median of 11.5 months postoperatively (IQR = 2–51). The reasons for these reoperations are as follows: (1) graft extrusion and hardware failure at an early stage (2 cases), (2) new onset C2–C3 degeneration with early signs of myelopathy, and (3) asymptomatic partial screw backout on routine follow-up imaging (Table 4). The median clinical follow up time for these patients was 40 months (IQR = 17.5–58.75), ranging from 15 to 60 months.

**Table 4:** Patients with four-level ACDF requiring revision surgery.

Levels	Age at surgery (yrs)	Gender	Diagnosis	Procedure	Time elapsed after index surgery (months)
C3–C7	66	Female	New onset degeneration C2–C3 level with early signs of cervical myelopathy	Posterior decompression and fusion C2–C4	61
C3–C7	55	Male	Partial back out of the C3 left screw	Removal of left C3 screw and replacement	21
C3–C7 (with C5 corpectomy)	67	Male	Pull out of the anterior cervical graft at the C3–4 and partial pull out of the fixation screws at C4 with shifting of the corpectomy graft	Revision anterior cervical decompression reconstruction C3–7 with removal ant fixation plate, repeat C3–4 anterior cervical discectomy and fusion, repositioning C4–6 corpectomy graft and C6–7 ant strut graft. Posterior C3–Th1 decompression and instrumented fusion	2
C3–C7	77	Female	Hardware failure with dislodgement of anterior cervical fixation system and new onset cervical kyphosis	Removal of the anterior fixation and posterior instrumentation, repeat bone grafting C3–C7. Posterior C2–C5 decompression and C2–T1 fixation	2

*ACDF* anterior cervical discectomy and fusion.

**Figure 3:**
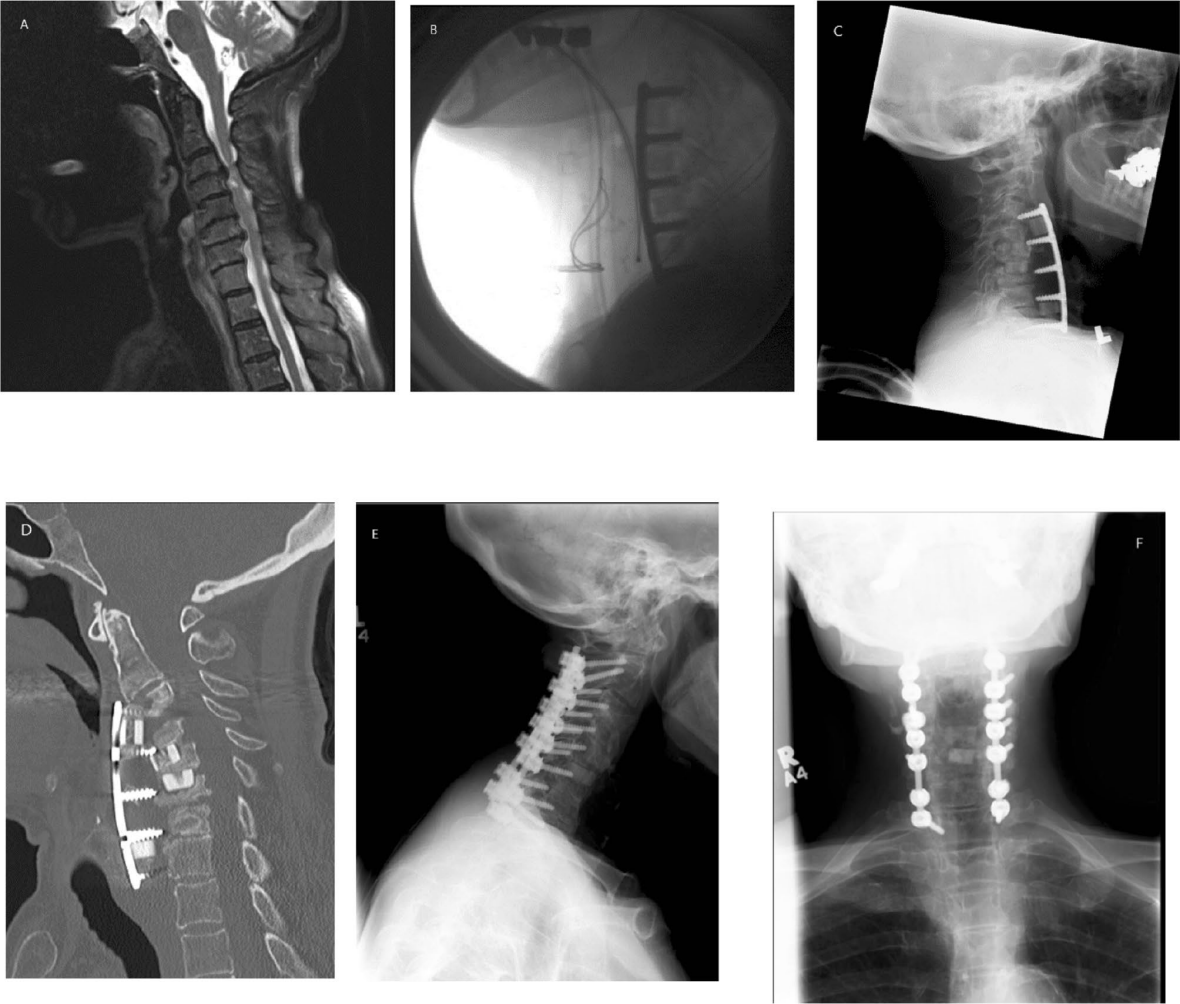
A 77-year-old female who presented with severe progressive myelopathy. A MRI scan of the cervical spine showed significant multilevel stenosis and cord compression (**A**). The patient underwent a four-level ACDF at the levels C3–C7 with good result (**B**). She was seen on a routine 8-week postoperative visit. The patient had no specific complaints and no swallowing dysfunction. She was improving neurologically. Lateral X-ray films showed complete dislodgement of the anterior hardware at all levels with the new onset of kyphosis (**C**,**D**). The patient underwent urgent revision with removal of previous anterior fixation hardware, repeat bone grafting C3–C7 and posterior C2–C5 decompression and C2–Th1 instrumented fusion (**E**,**F**). The ultimate outcome was excellent with significant resolution of symptoms.

Figure 3 demonstrates an illustrative case of a patient who required revision surgery.

## Discussion

Few studies have reported on clinical outcomes after multilevel ACDF. The current study demonstrated a significant improvement in cervical lordosis, NDI and VAS for neck pain scores in patients treated surgically with four-level ACDF due to cervical spondylosis.

It is known that cervical sagittal malalignment secondary to degenerative changes can lead to pain, spinal cord compression and the development of myelopathy^18,23^. Thus, restoring the sagittal profile is of crucial importance as it has been shown to be associated with improved clinical outcome scores and decreased rates of adjacent segment degeneration^24^. In the present study, cervical lordosis was assessed as a parameter of sagittal profile and the results revealed a significant improvement at the median 23 months follow-up. Similar results were also reported in a recently published retrospective study by Li et al^25^; in a cohort of 70 consecutive patients with four-level cervical spondylotic myelopathy, treated surgically with either anterior cervical corpectomy and fusion or anterior cervical decompression and fusion, the authors found a significant improvement in cervical lordosis after surgery.

Recent studies have shown improvement in clinical outcomes after four-level ACDF^11,15,17^. Wang et al^17^ reported satisfactory clinical outcomes with improvement in NDI, Neck and Arm pain and Japanese Orthopaedic Association (JOA) scores in a retrospective review of 32 patients who underwent four-level ACDF and had a minimum of 5 year follow up. Laratta et al^16^ found a significant improvement in NDI, Neck pain and Arm pain at 2-year follow up in a retrospective analysis of 46 patients with symptomatic spondylosis. Our results are in line with these studies; we demonstrated that four-level ACDF surgery may provide a significant clinical improvement in patients with multilevel cervical spondylosis. We consider these findings of importance, given the fact that NDI and VAS Neck pain have been shown to be predictors of satisfaction two and five years following anterior spine surgery^26^.

Although we found an improvement in VAS arm pain score, this difference was not statistically significant. There may be a couple of reasons for this finding. Firstly, patients with predominant radiculopathy represented the minority in this cohort. While all patients with symptomatic spondylosis are expected to have relief of their arm pain symptoms after adequate surgical decompression^17^, patients with predominant radiculopathy are more likely to have relief of their arm symptoms^27^. Secondly, the duration of symptoms that may have an impact on outcome was not investigated in this study. Recently, Tetrault et al^28^ showed that increased duration of symptoms correlates with outcomes in patients with cervical myelopathy. In the setting of predominant cervical radiculopathy, the impact of longer duration of symptoms on clinical outcome has also been demonstrated. A recent study by Burneikiene et al^29^ reported that patients with cervical radiculopathy who underwent 1 to 2 level ACDF surgery within 6 months of onset of symptoms demonstrated significantly greater reductions in VAS arm pain scores compared to those with symptoms for more than 6 months. Similarly, Tarazona et al^30^, in a retrospective analysis of 216 patients who underwent ACDF for radiculopathy, demonstrated that symptom durations of more than 2 years were predictive of higher neck and arm pain as compared to symptom durations of less than 6 months.

Although reports on pseudarthrosis rates after multilevel ACDF vary considerably in the literature, the rate of 14% demonstrated in this study is in line with previous reports. Bolesta et al^10^ demonstrated a pseudarthrosis rate of 53% among 15 patients treated surgically with three and four-level ACDF. More recently, Kreitz et al^15^ reported a 31% rate of radiographic pseudarthrosis in a retrospective analysis of 25 patients who underwent four-level ACDF. Contrary to these findings are the results reported by De la Garza-Ramos et al^12^; In this retrospective analysis of 71 patients who underwent three-level ACDF and 26 patients who underwent four-level ACDF, the pseudarthrosis rate was 5.6% and 15.4%, respectively. Similarly, Wang et al^17^ reported a pseudarthrosis rate of 6% in a study of 32 patients undergoing four-level ACDF.

In the present study, there were no significant differences in patient reported outcome measures between patients with and without pseudarthrosis. Recently published studies showed that, despite a high radiographic pseudarthrosis rate, patients who underwent four-level ACDF may achieve significant improvement in clinical outcomes with a low revision rate^15,31^. Our results are in agreement with these studies. Nevertheless, our results should be interpreted with caution given the small size of our cohort. A larger cohort study is necessary to address this knowledge gap.

Dysphagia and hoarseness are commonly observed in the early postoperative period after anterior cervical spine surgery^8,32^. Its incidence varies considerably and has been reported to be between 1–79%^11,12,14,33^. Moreover, the incidence of these complications increases with the number of ACDF levels performed^12,13,34^ due to a more extensive soft tissue exposure and swelling^35^. Preventative strategies such as reduced endotracheal tube cuff pressure^36^, dynamic surgical retraction^37^, use of local steroids in the retropharyngeal region^38^ and appropriate surgical dissection^39^ have been reported to reduce the incidence of these complications. Although symptoms are transient in the majority of cases and resolve within 6 months after surgery^40^, dysphagia may persist 6–24 months postoperatively in about 5–7% of cases^33,40^. Postoperative dysphagia and hoarseness were the most common complications observed in this study with an incidence of 32% and 14%, respectively. Our results are in line with previous reports in the literature^11,12,14^.

Overall, 4 patients (14%) required revision surgery; 2 out of 4 at an early stage due to graft extrusion and hardware failure. While ACDF has been shown to be an effective technique for preserving stability and lordosis of the cervical spine^41^, many authors have raised concerns on the efficacy of ACDF to achieve an adequate decompression in patients with multilevel spondylosis^8,42^; in such cases, a significant endplate resection or cervical corpectomy may be needed. However, a more aggressive decompression can be challenging, especially in elderly patients with comorbidities^43^ and low bone mineral density^44^, as it has been shown to be associated with a higher incidence of graft displacement or extrusion^41,45^; especially at early stages after the primary operation^43^. In these cases, anterior–posterior fusion can be performed depending on the clinical situation and determined on a case-by-case basis^46^. Nevertheless, in the absence of high-quality prospective studies, the impact of the addition of posterior fusion on clinical outcomes is still unexplored. Interestingly, none of the revisions were due to pseudarthrosis. This is not surprising given the fact that in many cases pseudarthrosis can be asymptomatic^15^. Nevertheless, it has to be pointed out that the true significance of asymptomatic pseudarthrosis on revision rate has not been investigated given the short follow up time of this cohort. Future studies with long term follow up could address this question.

In the current study, only one patient required further surgery due to adjacent segment disease, 61 months after index surgery. Adjacent segment disease may be a concern after ACDF surgery^47^. With an incidence of 2.9% annually it may affect more than 25% of all patients within ten years after index surgery^48^. While multilevel ACDF has been demonstrated to have a higher revision rate compared to single level cervical fusion, the risk of developing ASD has been shown to be significantly lower^48,49^. It seems that multilevel arthrodesis may have a protective effect against adjacent segment degeneration. However, given the small size of our cohort, limited evidence regarding the incidence of ASD after multilevel ACDF can be provided by the current study.

There are some limitations to this study. First, it is retrospective in nature with a small number of patients and therefore results should be interpreted with caution. Secondly, all procedures were performed in a single center by two surgeons which may limit the generalizability of the results. There was no specific patient reported instrument used for the assessment of postoperative dysphagia. Resolution of the symptoms was based on clinical examination and patient history during the follow-ups. Further, bone fusion was assessed predominantly with X-rays as CT scans were not used routinely in follow-up. Finally, the follow-up time in this study may not be sufficient to capture longer-term outcomes after surgery.

## Conclusion

This study showed improved clinical outcomes following four-level ACDF in patients with multilevel cervical spondylosis as compared to preoperative values. However, healthcare providers should be aware of the higher pseudarthrosis and reoperation rates as demonstrated in this study.
